# Prevalence and influential factors of isolated hepatitis B core antibody positivity in a Chinese adult population

**DOI:** 10.1038/s41598-023-50907-6

**Published:** 2024-01-06

**Authors:** Chengwei Wang, Xiaoqin Li, Chuanmeng Zhang, Li Xiao, Jianchun Xian

**Affiliations:** 1 Department of Hepatology, The Affiliated Taizhou People’s Hospital of Nanjing Medical University, Taizhou, Jiangsu Province China; 2 Central Laboratory, The Affiliated Taizhou People’s Hospital of Nanjing Medical University, Taizhou, Jiangsu Province China

**Keywords:** Hepatitis B, Viral infection

## Abstract

Isolated anti-HBc (IAHBc) is defined by the presence of anti-HBc in the absence of HBsAg and hepatitis B surface antibody (anti-HBs). IAHBc is of great clinical significance as a specific pattern of HBV infection, but IAHBc has not been fully clarified. This study aimed to explore the prevalence and influential factors of IAHBc from routine examination results of inpatients.A total of 61,247 individuals were included in the study, with a median age of 55 years (range: 43–68), and a male-to-female ratio of 0.90:1. The prevalence of current HBV infection (HBsAg positive) was 6.82%, while the prevalence of previous HBV infection (HBsAg negative but anti-HBc positive) was 48.63%. The prevalence of IAHBc was 12.31%. Among them, the rates for males were 7.10%, 52.16%, and 13.70%, respectively, which were significantly higher than the rates for females at 6.56%, 45.45%, and 11.06% (P < 0.05). The prevalence rates mentioned above were significantly reduced after vaccination (P < 0.05). The prevalence of IAHBc increases with age, rising from 0.23% in the age group of 15–29 years to 13.57% in individuals aged 80 and above. After the age of 50, the prevalence of IAHBc closely parallels the previous infection rate but shows no significant association with the current infection rate (P > 0.05). Among IAHBc individuals, approximately 33.83% tested positive for anti-HBe, and their anti-HBc absorbance values were significantly higher compared to anti-HBe negative individuals (7.08 and 5.31, P < 0.01). The prevalence of anti-HBe positivity among IAHBc individuals does not vary with changes in the previous infection rate and age (P > 0.05).

## Introduction

HBV infection remains a significant global public health issue. According to the World Health Organization (WHO), in 2019, approximately 296 million people worldwide were infected with the HBV, and approximately 1 million deaths occur each year due to HBV-related diseases, including liver cancer^1,2^. In China, there are approximately 86 million people infected with HBV, of which about 28 million are chronic hepatitis B (CHB) patients. In 2020, there were 830,000 deaths from liver cancer globally, with China accounting for 391,000, or 47% of the total^3^. HBV core antigen (HBcAg) has stronger immunogenicity compared to HBV surface antigen (HBsAg), therefore, it is believed that HBcAg antibodies (anti-HBc) are produced earlier and have a longer duration compared to anti-HBs, which have a more significant importance in both current and previous HBV infection. Anti-HBc positive individuals with current infections are often co-existent with HBsAg, while anti-HBc positive individuals with previous infections often co-exist with anti-HBs. However, due to factors such as host, virus, or detection sensitivity, a subset of individuals may only exhibit IAHBc positivity with both HBsAg and anti-HBs negative. Such individuals have received attention due to their significance in occult HBV infection (OBI) and HBV reactivation^4–6^. However, there is limited research on the prevalence and associated factors of IAHBc, which hinders a proper understanding of this pattern in clinical practice. This study aims to explore the epidemiological characteristics and influential factors of IAHBc based on routine examination results of hospitalized patients, providing a theoretical basis for a better understanding of the significance of IAHBc in the clinical setting^7^.

## Materials and methods

### Research object

The study is a retrospective, single-center study conducted in 2021 at Taizhou People's Hospital in Jiangsu, China. The study aimed to analyze the HBV serological and immunological markers of hospitalized patients in the non-infectious disease department (which lacks representation of HBV-infected patients due to their concentration in the infectious disease department) and non-pediatric department (where routine HBV marker screenings are not performed).

Inclusion criteria: (1) Age > 14 years old; (2) Non-infectious or hepatic disease department hospitalized patients; (3) Patients who underwent four examinations including HBV-M, anti-HIV, anti-HCV, and anti-TP upon admission.

Main exclusion criteria: (1) Repeat patients; (2) Patients with pending confirmation for anti-HIV, positive results for anti-HCV and anti-TP; (3) Patients with missing identification card numbers or examination data that may affect uniqueness and result determination.

All participants remained anonymous throughout the study. The study was approved by the Ethics Review Committee of Taizhou People’s Hospital (approved number: KY2022-092-01). This study was a retrospective study, the data was the routine examinations of patients, and the patient's privacy was not involved, so informed consent was exempted. All methods were performed in accordance with relevant guidelines and regulations.

### Data collection

Data collection in this study was obtained through the Hospital Laboratory Information System (LIS), which provided relevant information about the cases. This included demographic characteristics of the cases (gender, age, identification number), department of visit, and results of pathogen testing (HBV, HIV, HCV, and syphilis serological tests).

### Laboratory test methods

All serological tests were conducted in the central laboratory of Taizhou People’s Hospital within 24 h of specimen collection. The HBV serological markers [HBsAg, anti-HBs, hepatitis B e antigen (HBeAg), hepatitis B e antibody (anti-HBe), anti-HBc] of the patients were detected using the Abbott i2000SR fully automated immunoassay analyzer and its corresponding reagent kits (ARCHITECT) with chemiluminescent microparticle immunoassay (CMIA) technology. The anti-HIV, anti-HCV, and anti-TP tests were performed using the Roche Diagnostics Elecsys^®^ Immunoassay analyzer LiCA500, with reagent kits purchased from Boyang Biotech (Shanghai) Co., Ltd., and results were interpreted following the manufacturer's instructions.

### Definition of related terms

Here are the definitions of the terms based on the serological immunological results of HBV-M and their occurrence frequency:

1. Current infection: HBsAg positive ( +).
2. Previous infection: HBsAg negative but anti-HBc positive individuals.
3. IAHBc: HBsAg negative and anti-HBs negative but anti-HBc positive.
4. Infected individuals: Previous infection and current infection.

### Grouping by age and China strategies of hepatitis B vaccination

The age groups based on the changing hepatitis B vaccine immunization strategies in China:

- Individuals aged 20 to 29 years, born between 1992 and 2001 (inclusive)—This group corresponds to the period when hepatitis B vaccination was initially included in the immunization program (also the catch-up vaccination period carried out between 2009 and 2011). Individuals aged 15 to 19 years, born in or after 2002—This group corresponds to the period when hepatitis B vaccination was officially included in the immunization program in 2002. The above population is divided into the group of individuals < 30 years old, that is, the group of individuals born after vaccination.
- Individuals aged ≥ 30 years old, born before 1991—This group corresponds to the period before the implementation of the immunization program. This group is the population born before vaccination.
- The other grouping is conducted in 10-year-old intervals.

### Statistical analyses

This study utilized SPSS 26.0 for data analysis. For quantitative data that follows a normal distribution, comparisons were made using the independent samples t-test, and the results were presented as x ± s (mean ± standard deviation). For quantitative data that doesn't satisfy the normality assumption, comparisons were made using the Mann–Whitney U test, and the results were presented as M_d_ (Q1-Q3) (median with interquartile range). For qualitative data, frequencies were presented, and comparisons were made using the chi-square test. A significance level of P < 0.05 was considered statistically significant.

### Ethics approval and consent to participate

The study was approved by the Ethics Review Committee of Taizhou People’s Hospital (KY2022-092-01). This study was a retrospective study, the data was the routine examinations of patients, and the patient's privacy was not involved, so informed consent was exempted.

## Results

### The basic characteristics of the study population

In 2021, a total of 79,891 hospitalized patients were extracted from the Hospital LIS system. Out of these, 3176 cases had missing or unknown identification information, 10,851 cases were repeat admissions, 2263 cases were from the infectious disease and hepatology department, 817 cases were pediatric patients (< 15 years old), 150 cases tested positive for anti-HCV, 20 cases had pending confirmation for anti-HIV, and 1367 cases tested positive for anti-TP, were excluded from the study. Finally, a total of 61,247 cases met the inclusion criteria.

Within the study population, the male-to-female ratio was 0.9:1. The majority of individuals were of Han ethnicity (99.6%). The age of the participants ranged from 15 to 101 years, with a median age of 55 years (15–101). The age distribution showed that individuals aged 15 to 29 accounted for 9.41%, 30 to 40 accounted for 12.18%, 41 to 50 accounted for 15.23%, 51 to 60 accounted for 23.79%, 61 to 70 accounted for 19.69%, 71 to 80 accounted for 14.65%, and those above 80 accounted for 5.05%. This indicates that the study population has a skewed distribution with a higher proportion of older individuals.

### The impact of gender and vaccination status on HBV infection and the prevalence of IAHBC

Among the 61,247 cases that met the inclusion criteria, the current HBV infection rate was 6.82%, the previous infection rate was 48.63%, and the rate of IAHBc positivity was 12.31%. Among males, the rates were 7.10% for current infection, 52.16% for previous infection, and 13.70% for IAHBc, which were significantly higher than the rates among females (6.56% for current infection, 45.45% for previous infection, and 11.06% for IAHBc) (P < 0.05). Individuals born after vaccination had a significantly lower HBV infection rate (P < 0.05). Please refer to Tables 1 and 2 for details.

**Table 1:** The effect of gender on HBV infection and IAHBc prevalence.

	n	Age (year) M_d_ (Q_1_-Q_3_)	Current infection [n, (%)]	Previous infection [n, (%)]	IAHBc [n, (%)]
Total	61,247	55 (43–68)	4175 (6.82)	29,783 (48.63)	7540 (12.31)
Male [n, (%)]	28,985 (47.32)	59 (48–70)	2057 (7.10)	15,120 (52.16)	3972 (13.70)
Female [n, (%)]	32,262 (52.68)	53 (39–65)	2118 (6.56)	14,663 (45.45)	3568 (11.06)
z/χ^2^		42.56	6.80	275.60	98.89
P value		< 0.001	0.009	< 0.001	< 0.001

**Table 2:** The effects of vaccination on HBV infection.

	n	Current infection [n, (%)]	Previous infection [n, (%)]	IAHBc [n, (%)]
Total	61,247	4175 (6.82)	29,783 (48.63)	7540 (12.31)
Individuals born before vaccination (≥ 30 years old) [n, (%)]	55,486 (90.59)	4060 (7.32)	29,520 (53.20)	7527 (13.57)
Individuals born after vaccination (< 30 years old) [n, (%)]	5761 (9.41)	115 (2.00)	263 (4.57)	13 (0.23)
z/χ^2^		232.63	4942.25	860.34
P value		< 0.001	< 0.001	< 0.001

Current infection: HBsAg positive ( +). Previous infection: HBsAg negative but anti-HBc positive individuals. *IAHBc* HBsAg negative and anti-HBs negative but anti-HBc positive.

### The impact of age on HBV infection and the prevalence of IAHBC

**Figure 1:**
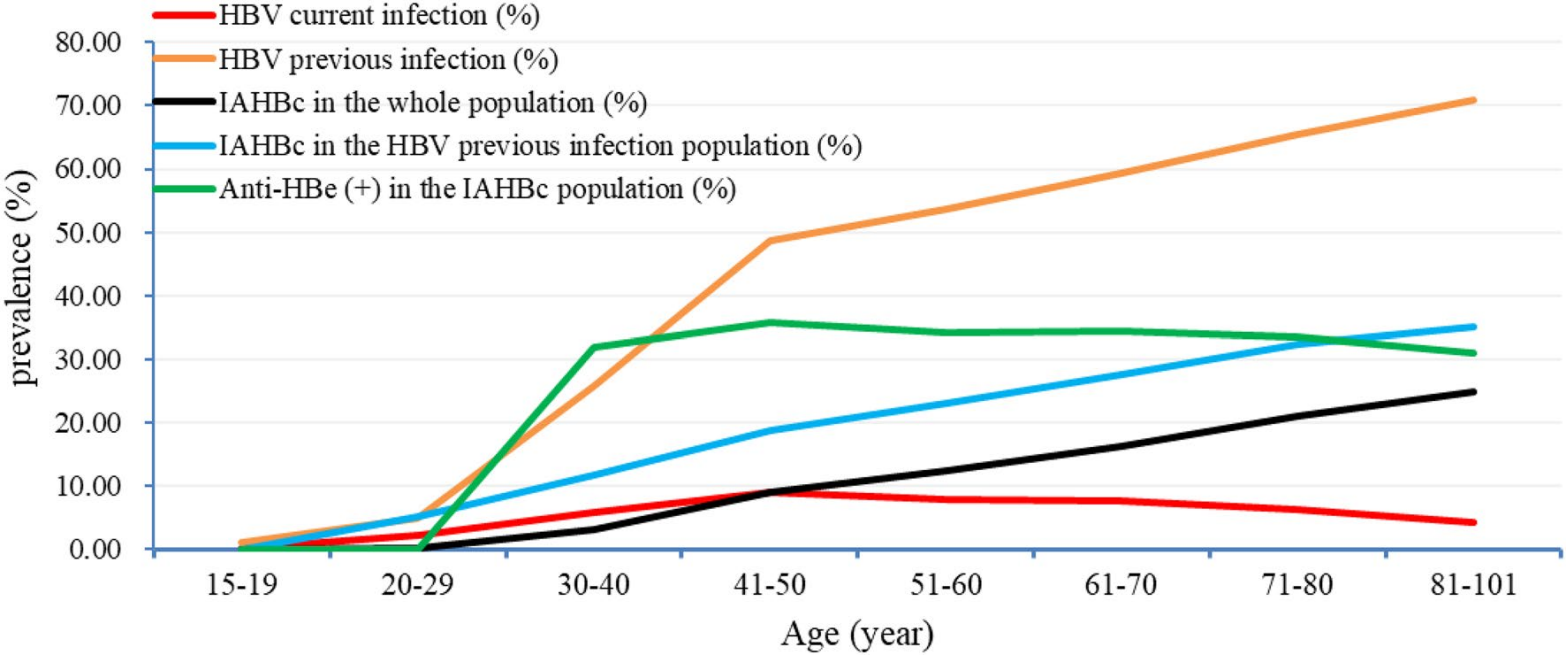
The prevalence and age distribution of different patterns of HBV infection.

The previous infection rate of HBV, prevalence of IAHBc, and the rate of IAHBc among previous infection cases all increase with age, but exhibit a nonlinear relationship. Based on their increasing rates, three stages can be identified. In the population born after vaccination (15–29 years old), the rates show a slow and gradual increase at a lower level. In individuals born before vaccination, there is a rapid increase in these rates during the age range of 30–50 years. For individuals aged 50 and above, the rates of HBV previous infection, IAHBc, and the positivity rate of IAHBc among individuals with previous infection show a parallel linear increase with age. On the other hand, the current infection rate of HBV gradually increases with age until around 41–50 years, after which it slowly declines. Please refer to Table 3 and Fig. 1 for details.

**Table 3:** The effects of age on HBV infection and the prevalence of IAHBC.

Age	n (%)	Current Infection [n, (%)]	Previous Infection [n, (%)]	IAHBc [n, (%)]
15–19	682 (1.11)	2 (0.29)	8 (1.17)	0
20–29	5079 (8.29)	113 (2.22)	255 (5.02)	13 (0.26)
30–40	7460 (12.18)	445 (5.97)	1933 (25.91)	228 (3.06)
41–50	9329 (15.23)	840 (9.00)	4548 (48.75)	850 (9.11)
51–60	14,572 (23.79)	1142 (7.84)	7836 (53.77)	1807 (12.40)
61–70	12,060 (19.69)	930 (7.71)	7142 (59.22)	1979 (16.41)
71–80	8972 (14.65)	569 (6.34)	5870 (65.43)	1895 (21.12)
81–101	3093 (5.05)	134 (4.33)	2191 (70.84)	768 (24.83)
z/χ^2^		365.39	8342.36	2741.77
P value		< 0.001	< 0.001	< 0.001

### The relationship between IAHBc and HBV infection

The prevalence of IAHBc is not significantly related to the current infection rate (P > 0.05), but it is significantly associated with the previous infection rate (P < 0.05). The proportion of IAHBc in the overall population and among individuals with previous infection increases with age. It rises from 0.23% and 4.94% in the age group of 15–29 years to 13.57% and 35.05% in those aged 80 and above. After the age range of 41–50 years, the rate of IAHBc positivity in the overall population, the rate of IAHBc among previous infection cases, and the previous infection rate exhibit a parallel trend. Please refer to Fig. 1 for details.

### A comparison of age, gender, and anti-HBc titer between anti-HBe (+) and anti-HBe (−) IAHBc individuals

Further analysis of the anti-HBe status among IAHBc individuals revealed that approximately one-third (33.83% in our dataset) tested positive for anti-HBe. This proportion remained consistent among individuals born before vaccination, regardless of variations in previous infection rates and age (P > 0.05), as shown in Fig. 1. The mean age for anti-HBe positive IAHBc individuals was 64.11 ± 12.58 years, while for anti-HBe negative IAHBc individuals was 64.61 ± 12.88 years. The gender ratio (male to female) for both groups was 1.12 and 1.11, respectively, and there were no significant differences between the two groups (P > 0.05). However, the anti-HBc titer (qualitative result) for anti-HBe positive IAHBc individuals was significantly higher compared to anti-HBe negative individuals (7.08 vs 5.31, P < 0.01). Please refer to Table 4 for details.

**Table 4:** Comparison of age, gender and anti-HBc titer between anti-HBe (+) and anti-HBe (−) IAHBc individuals.

	n	Age (year) M_d_ (Q_1_–Q_3_)	Gender			anti-HBc titer (s/co) M_d_( Q_1_–Q_3_)
			Male	Female	M/F	
anti-HBe (−)	4989	66 (55–74)	2623	2366	1.11	5.31 (3.18–6.81)
anti-HBe (+)	2551	65 (54–74)	1349	1202	1.12	7.08 (5.98–7.95)
z/χ^2^		− 1.85	0.06			33.23
P value		0.064	0.801			< 0.001

*Anti-HBe* Hepatitis B e antibody, *anti-HBc* Hepatitis B core IgM and IgG antibodies, *IAHBc* Isolated anti-HBc, *M/F* male to female ratio.

## Discussion

Our data indicates that although HBV infection rates (including current and previous infection) have significantly decreased after vaccination (6.56% and 60.52% respectively), there remains a higher prevalence among individuals born before vaccination. Furthermore, the infection rates gradually increase with age, particularly for previous infection rates, which rise rapidly from 4.57% in the age group of 15–29 years to 54.17% in the age group of 45–49 years, and then slowly increase to 70.84% in individuals above 80 years of age. In contrast, the HBsAg positive rate shows a rapid increase until the age group of 45–49 years, rising from 2.00% in the age group of 15–29 years to 9.45%, and then slowly declining to 4.59% in individuals above 80 years of age.

IAHBc was a significant focus of this study, and the results showed that the IAHBc rate accounted for 12.31% of the total study population and 25.32% of the previous infection cases. The IAHBc rate increased with age, from 0.23% and 5.0% in the age group of 15–29 years to 24.83% and 35.05% in individuals above 80 years of age. IAHBc accounted for 31.19% (7540 out of 24,172) of individuals who tested negative for both HBsAg and anti-HBs (24,172, representing 39.47% of the total population). The prevalence of IAHBc showed a relative stable linear parallel increase with age, independent of current infection rates and gender. These findings suggest that after HBsAg seroconversion, the majority of individuals develop anti-HBs, while IAHBc represents only a minority (approximately 1/4) of individuals with previous infection, especially in the early stages of seroconversion and in younger ages.

The prevalence of IAHBc in the population can vary significantly, depending on factors such as the local HBV epidemicity and the presence of co-infections. However, there seems to be some patterns to its prevalence. For example, in high- to medium-endemic areas, the prevalence of IAHBc in the general population can range from 5.9 to 11.9%^6,8,9^, while in low-endemic areas like Europe and North America, it is typically between 1 and 4% (in populations with HBV serological rates of 10% to 20%)^10^. In individuals with HBV infection or past infection, the prevalence of IAHBc tends to remain relatively stable.

In our study, IAHBc accounted for 22.20% (7540/33,958) of HBV infections and 25.32% (7540/29,783) of previous infections. These findings are similar to those reported by Launay et al.^11^ (mean age of 47 ± 15 years) who found that IAHBc accounted for 24.83% (362/1458) of HBV infections. However, the proportion of IAHBc among previous infections was higher in this study (34.8%, 362/1039). This difference may be attributed to the inclusion of individuals with immunodeficiencies such as positive anti-HCV or anti-HIV antibodies, because these individuals had lower HBsAg conversion rates.

The prevalence of IAHBc in the population can vary depending on the specific criteria used for its measurement. It is important to be cautious when citing prevalence rates of IAHBc, as different studies may use different definitions. Some studies might mistakenly include all individuals who are positive for anti-HBc (including both those who are negative for HBsAg and positive for anti-HBc, as well as those who are positive for both HBsAg and anti-HBc), which could lead to an artificially inflated rate of IAHBc^12,13^.

For example, several influential articles have incorrectly cited a "34.1% overall prevalence rate of IAHBc in the Chinese population aged 1–59 years (in the 10th line of part 5 of reference 14 and in the 11th line of the second paragraph of part 1 of reference 15)^14,15^", while another review article (in the third line of the second paragraph of the “Introduction” section of reference 7) cited a prevalence rate of 32.0%^7^. However, the original text of the references annotated on these data in the above articles did not include these data. These discrepancies are likely the result of misinterpretation or misquotation.

In our study, we found that the prevalence of IAHBc increases with increasing previous infection rates and age. It gradually increases from 0.23% in the age group of 15–29 years to 24.83% in individuals aged 80 and above. The overall prevalence rate of IAHBc in our study population was 12.31%, which includes individuals from an age group (60 years and above) considered to be in a high-endemic region. Therefore, it is important to note that achieving an IAHBc rate exceeding 30% is unlikely, especially when studying younger age groups with a lower previous infection rate. Even in our study population over 80 years of age, with a previous infection rate of 70.84%, the IAHBc rate was only 24.83%.

This study further explored the pattern of anti-HBe positivity among IAHBc individuals and found that 33.51% tested positive for anti-HBe. The presence of anti-HBe positivity did not vary with age, gender, or previous infection rates. There were no significant differences in age and gender ratio between anti-HBe positive and negative IAHBc individuals. However, the anti-HBc titer was significantly higher in the anti-HBe positive group compared to the anti-HBe negative group (P < 0.001). The association between the anti-HBc titer and liver tissue inflammation activity and the presence of covalently closed circular DNA (cccDNA) is well-documented^5,16,17^. Additionally, the expression of anti-HBe indicates a strong cellular immune response and immunological recovery^18^. Therefore, it can be considered that individuals with anti-HBe positivity may be in a partial immune clearance stage. The detection rate of OBI in the anti-HBe positive group (15.61%, 32/205) was significantly higher than that in the anti-HBe negative group (3.73%, 10/268) (P < 0.05)^19^. This suggests that even among IAHBc individuals, it is possible to further classify them into two different states based on the presence or absence of anti-HBe, which may have different clinical implications.

IAHBc is a special serological pattern that can occur due to various reasons or backgrounds, such as the HBV infection window period, false positivity, "S" region variants, or immunodeficiency^7,17^. Currently, there is no precise and reliable method for distinguishing the underlying cause of IAHBc. HBV DNA quantification and IgM anti-HBc testing can provide some value in distinguishing HBV variants and acute infection window periods. However, further longitudinal follow-up or retesting with different methods, along with the confirmation of other markers such as HBeAg and anti-HBe, are necessary^21^.

Among the 7540 IAHBc cases in this study, only one elderly male cancer patient had low-level positive HBeAg (HBV DNA testing was not performed), and there was no case of positive IgM anti-HBc. Considering China's high endemicity of HBV, it suggests that IAHBc cases caused by HBV "S" region variants, acute infection window periods, or false positivity may be very rare. Instead, most cases may be attributed to late immunity^10^, as the titer of anti-HBs gradually decreases over time and as age increases after HBsAg seroconversion, which has been confirmed by other studies^11,20^.

Therefore, in the general population, IAHBc is a significant risk factor for serological OBI, particularly in anti-HBe positive IAHBc individuals. If the focus is on OBI, it is important to further examine the anti-HBe status. Studies, such as Cai et al.^19^, have shown that OBI has the highest detection rate in IAHBc cases where both anti-HBc and anti-HBe are positive (15.61%, 32/205). This aligns with the findings of this study, suggesting that if anti-HBe is still positive, it may indicate an early stage of partial immune activation and recovery. On the other hand, if anti-HBe is negative and immunosuppression-related IAHBc can be ruled out, this IAHBc positivity may be safer with a lower OBI detection rate^6,19^.

This study has several strengths, including a large sample size, a focused data collection period, and the use of reliable testing methods. The participants were unaware of their HBV status at the time of screening, which increases the reliability of the results. However, there are some limitations to consider. Firstly, the participants may not represent the entire population of Taizhou. Although the participants were a heterogeneous group in terms of demography, they were limited to hospitalized patients aged 15 and above, which skews towards an older age group and does not include the transient population (which accounts for around 10% of the total population of Taizhou). Secondly, the study did not include patients from the department of infectious diseases and hepatology, where the HBV infection rate may be relatively high. This could have an impact on the overall prevalence rate. Additionally, the study did not have a follow-up period and did not include HBV DNA, liver function results, or related examinations. This could limit the determination of disease status and natural history staging.

## Conclusion

This cross-sectional study provides insights into the prevalence and factors influencing IAHBc positivity. The results highlight a significant reduction in HBV infection rates among individuals born after the implementation of the HBV vaccination program. The prevalence of IAHBc among the general hospitalized population was found to be 12.31%, accounting for 25.32% of past infections. The prevalence of IAHBc increased parallel to the past infection rate with age, and the prevalence was higher in males than in females. Among IAHBc individuals, approximately one-third tested positive for anti-HBe, and their anti-HBc titers were significantly higher compared to those who tested negative for anti-HBe, irrespective of variations in past infection rates and age. These findings contribute to a better understanding of the clinical significance of IAHBc.

## Data Availability

All data generated or analysed during this study are included in this article.
