# Author Correction: Prevalence and influential factors of isolated hepatitis B core antibody positivity in a Chinese adult population

**DOI:** 10.1038/s41598-024-53273-z

**Published:** 2024-02-01

**Authors:** Chengwei Wang, Xiaoqin Li, Chuanmeng Zhang, Li Xiao, Jianchun Xian

**Affiliations:** 1 Department of Hepatology, The Affiliated Taizhou People’s Hospital of Nanjing Medical University, Taizhou, Jiangsu Province China; 2 Central Laboratory, The Affiliated Taizhou People’s Hospital of Nanjing Medical University, Taizhou, Jiangsu Province China

Correction to: *Scientific Reports* 10.1038/s41598-023-50907-6, published online 06 January 2024

The original version of this Article contained an error in the Discussion section.

As a result,

“However, the proportion of IAHBc among previous infections was higher in this study (34.8%, 362/1.39).”

now reads:

“However, the proportion of IAHBc among previous infections was higher in this study (34.8%, 362/1039).”

The original Article has been corrected.

